# Identifying barriers and facilitators of the inclusion of pregnant individuals in hepatitis C treatment programs in the United States

**DOI:** 10.1371/journal.pone.0277987

**Published:** 2022-11-18

**Authors:** Lynn M. Yee, Seema K. Shah, William A. Grobman, Patricia Z. Labellarte, Leonardo Barrera, Ravi Jhaveri

**Affiliations:** 1 Division of Maternal-Fetal Medicine, Department of Obstetrics and Gynecology, Northwestern University Feinberg School of Medicine, Chicago, Illinois, United States of America; 2 Division of Advanced General Pediatrics, Department of Pediatrics, Northwestern University Feinberg School of Medicine, Chicago, Illinois, United States of America; 3 Smith Child Health Outcomes Research and Evaluation Center, Ann and Robert H. Lurie Children’s Hospital, Chicago, Illinois, United States of America; 4 Division of Maternal-Fetal Medicine, Department of Obstetrics and Gynecology, The Ohio State University School of Medicine, Columbus, Ohio, United States of America; 5 Division of Infectious Diseases, Department of Pediatrics, Northwestern University Feinberg School of Medicine, Chicago, Illinois, United States of America; Johns Hopkins University School of Medicine, UNITED STATES

## Abstract

**Background:**

The rising prevalence of hepatitis C virus (HCV) infection and the availability of direct acting antivirals for HCV treatment has prompted a public health goal of HCV eradication. Despite the availability of treatment for HCV, treatment programs have generally excluded pregnant individuals. Our objective was to query patients and clinicians to identify barriers to including pregnant individuals in HCV treatment programs.

**Methods and findings:**

This qualitative investigation included obstetricians and previously/currently pregnant individuals with HCV. Participants completed interviews regarding knowledge of and attitudes towards HCV treatment and perceived barriers to treatment during pregnancy. Data were analyzed using the constant comparative method. Obstetricians (N = 18) and patients (N = 21) described concerns about equity, access, and cost. Both expressed uncertainty about safety and confirmed a need for clinician education. Obstetricians emphasized the lack of professional guidelines. Although some clinicians expressed concern about patient adherence and engagement, patients were largely desirous of treatment; both groups identified potential benefits of antenatal treatment.

**Conclusions:**

Both patients and obstetricians were generally receptive to HCV treatment in pregnancy and recognized pregnancy as an important window of opportunity for treatment. Our findings suggest the need for further research on maternal-fetal safety of HCV treatment as well as on interventions to ensure fair and appropriate access to HCV treatment for pregnant individuals.

## Introduction

In parallel with ongoing opioid epidemic in the United States (US), hepatitis C virus (HCV) prevalence continues to rise, particularly among young adults under 40 [1, 2]. For childbearing individuals of reproductive age, the increase in HCV prevalence has driven significant increases in the prevalence of HCV among pregnant people, HCV perinatal transmission, and other sequelae of HCV during pregnancy, such as low birthweight or gestational diabetes mellitus [3–7]. As a result, in 2020, universal screening for HCV for all adults, including pregnant individuals, was recommended by the United States Preventive Services Task Force (USPSTF) and subsequently endorsed by the American College of Obstetricians and Gynecologists (ACOG) and the Society for Maternal-Fetal Medicine (SMFM) [8–11].

Globally, rising HCV prevalence has been accompanied by a public health strategy focused on eradication. Universal screening recommendations were designed to identify patients who could benefit from direct acting antiviral (DAA) medications, which can cure 97–99% of HCV patients after 8 to 12 weeks of treatment [12–14]. Micro-eradication programs have been implemented in many diverse settings with success, [15–17] and treatment programs have become less restrictive for non-pregnant individuals [18–20]. However, despite the importance of universal treatment, pregnant individuals have been largely excluded from eradication strategies. Safety and efficacy data for DAA use during pregnancy lags behind data from the general population, with reports only recently suggesting that DAA therapy is safe, well-tolerated, and efficacious when administered to pregnant individuals [21, 22]. Due to limited research on HCV therapy during pregnancy, professional recommendations regarding HCV treatment for this population specify treatment should only be undertaken in the context of research [11].

Despite limited data, there are compelling reasons why treating HCV during pregnancy could be an important part of the public health strategy to eradicate HCV [22]. Pregnant individuals have enhanced access to health care and the duration of treatment can be accomplished during the second and third trimester. Additionally, HCV cure during pregnancy may mitigate the perinatal risks associated with HCV, including perinatal transmission [22]. Yet, patient and clinician perspectives on HCV treatment during pregnancy are unclear [23–25]. Understanding the perspectives of these stakeholders regarding HCV treatment in this critical window of opportunity is essential to furthering the goal of HCV eradication. Thus, our objective was to examine patient and obstetrician attitudes about HCV treatment during pregnancy.

## Methods

### Patient participants

This prospective qualitative investigation examined the attitudes of pregnant or previously pregnant individuals with HCV and obstetric clinicians about HCV treatment during pregnancy. Patient participants with HCV were eligible for inclusion if they were known to have HCV infection (HCV antibody positive with detectable HCV RNA at any point in their medical history), regardless of treatment history or coinfection status, were age 18 to 45, and were able to complete an interview in English. Individuals who did not have a detectable HCV RNA in their medical records were not included, but participants may have achieved an undetectable viral load after treatment. Although nulliparity was not an exclusion criteria for participation, due to the goals of the study, individuals who were currently or previously pregnant were prioritized for recruitment. Patients were recruited via the electronic medical record census of the adult and obstetric infectious disease practices and through the institution’s data warehouse. Eligible participants were contacted by telephone about the study and provided written or verbal consent prior to participation. Patient interviews were conducted between April 13, 2020 and August 5, 2020.

### Obstetrician participants

Obstetrician participants were recruited via a review of personnel records at a large academic medical center and its affiliated community hospitals. Purposeful sampling was performed in order to recruit a diverse sample of participants, with regard to specialty, time since training, and practice type. Obstetric and gynecology specialists (Ob/Gyn) as well as maternal-fetal medicine (MFM) subspecialists were eligible to participate. All obstetrician participants were required to have completed all training (residency and, if applicable, fellowship) and to be actively involved in the care of pregnant patients. Experience with treatment of HCV was not a condition for inclusion, as our study goals included assessing the general knowledge and attitudes of obstetricians regardless of HCV treatment familiarity. Individuals were recruited via email, telephone, and face-to-face recruitment. Obstetrician interviews were conducted between February 21, 2020 and May 29, 2020. This study focused on obstetrician perspectives because these clinicians are typically the most common health care providers who interface with pregnant patients during routine care, and thus they may serve as the initial source of information for and treaters of pregnant patients with HCV.

### Interviews and analysis

Both patient and obstetrician participants underwent individual virtual interviews led by a trained research team member. The interviewer had extensive prior experience with qualitative interviewing, including the empathic, conversational style necessary to engage in discussions about sensitive topics such as HCV. The interviewer was neither a colleague/supervisor of obstetrician participants, nor a clinician involved in the care of patient participants. The interviewer conducted interviews using semi-structured interview guides for each respective participant type (S1 Appendix). Patient interview guides addressed knowledge of HCV and its treatment; attitudes towards therapies in general, and HCV treatment specifically during pregnancy; and experiences with, or perceived barriers to, HCV treatment during pregnancy. Obstetrician interview guides addressed clinical experience with HCV, knowledge and attitudes about HCV treatment during pregnancy, perspectives on the current state of evidence, opinions on balancing maternal and fetal interests during pregnancy, and practical issues regarding HCV treatment. All interviews were digitally recorded, professionally transcribed, and deidentified prior to analysis.

For both patient and obstetrician samples, the sample size was guided by the concept of saturation, or redundancy, in qualitative research [26]. Our *a priori* targets for recruitment were approximately 20 individuals in each group; saturation for patient participants was achieved after 21 participants and for providers was achieved after 18 participants.

Interview transcripts were analyzed by three study team members (PL, LB, LS) using a constant comparative approach to identify themes [27, 28]. Analysis was supported by Dedoose (Dedoose Version 9.0.46, web application for managing, analyzing, and presenting qualitative and mixed methods research data [2021]. Los Angeles, CA: SocioCultural Research Consultants, LLC www.dedoose.com), a web-based qualitative and mixed methods analysis application [29]. Analysis of obstetrician data occurred first. The study team first collaborated to create an initial codebook based on the obstetrician interview guides. In this process, three obstetrician interview transcripts were reviewed in-depth and discussed by three team members (PL, LB, LS) to finalize the codebook for analysis of obstetrician data. After this codebook was finalized, each of the 18 interview transcripts was coded by one of three team members (PL, LB, LS) and reviewed by one of two team members (PL, LB) as a secondary coder. All investigators met after primary and secondary coding to review their coding for each transcript to establish agreement, ensure reliability, and refine code definitions. Once all transcripts were coded and/or reviewed by both team members, all code excerpts were extracted and reviewed by one of two team members (PL, LS) to identify themes. Iterative team discussion was used to refine, collapse, and re-aggregate themes as data analysis was performed. The same constant comparative process was performed for analysis of patient data.

All participants provided written or verbal informed consent prior to participation, and all participants were offered a $50 gift card for their participation. The study was approved by the Institutional Review Boards of the Ann and Robert H. Lurie Children’s Hospital of Chicago and of Northwestern University. The Consolidated Criteria for REporting Qualitative research (COREQ) were reviewed and applied to this investigation.

## Results

From February through August 2020, 21 patients and 18 obstetricians participated in interviews. Of the patient participants, 15 had received HCV treatment, whereas 6 had never received treatment (Table 1). All 21 patients had a history of pregnancy or were pregnant at the time of the interview. Of the 18 obstetricians, 10 were MFMs and 8 were Ob/Gyns without subspecialty training (Table 1). In these analyses, emergent themes were categorized as barriers or facilitators to HCV treatment during pregnancy and classified by the participant type.

**Table 1:** Obstetrician and patient participant characteristics.

Characteristic	N (%), median (IQR), or mean ± SD
Obstetricians (N = 18)	
Age, years	48.5 (39, 58.2)
Race and ethnicity (self-identified)	
Non-Hispanic White	15 (83%)
Non-Hispanic Black	1 (6%)
Asian	2 (11%)
Time since completion of training, years	
0–5	6 (33%)
6–10	2 (11%)
11–20	3 (17%)
≥21	7 (39%)
Female	15 (83%)
Obstetrician type	
Obstetrician/gynecologist specialists	8 (44%)
Maternal-fetal medicine subspecialists	10 (56%)
Patients (N = 21)	
Age, years	37 (32, 41)
Race and ethnicity (self-identified)	
Non-Hispanice White	15 (71%)
Non-Hispanic Black	2 (9.5%)
Hispanic	2 (9.5%)
Asian	1 (5%)
Other	1 (5%)
Years since HCV diagnosis	14.3 ± 12.7
HCV treatment history	
None	6 (29%)
Direct-acting antivirals	11 (52%)
Interferon and ribavirin	2 (9.5%)
Interferon, ribavirin, and direct-acting antivirals	2 (9.5%)
Prior or current pregnancy	21 (100%)

### Obstetrician-identified barriers (Table 2)

Five overarching themes emerged from obstetrician interviews regarding barriers to HCV *treatment* during pregnancy: insufficient knowledge, inadequate evidence base, health inequities and access concerns, perceived patient burdens, and safety concerns.

**Table 2:** Obstetrician perceptions of barriers to treatment of HCV during pregnancy.

Theme	Subtheme	Examplary quotations
Insufficient knowledge	Lack of training regarding HCV in pregnancy	“I haven’t had training [on newer HCV treatment options] per se… I wouldn’t say I’m super facile with the data, but I know the broad concept…(MFM)
	Misconceptions and incorrect knowledge	“I could be wrong but the frequency of infusions [may be a barrier to initiating Hep C treatment for pregnant patients]. I don’t know how frequent that is, but just in general a lot of barriers of patients are their ability to get to the clinic as frequently as we need them to in pregnancy.” (MFM)
	Infrequent exposure to patients with HCV	“I think one barrier is just that most of us it’s clear you could…don’t have a ton of experience with hepatitis and so they’re wary about treating a disease they don’t have a lot of experience with.” (MFM)
Inadequate evidence base and uncertainty about safety	Lack of professional guidelines	“I would go by whatever guidelines ACOG guidelines, maternal fetal medicine are out there.” (Ob/Gyn)
	Lack of pregnancy-specific safety data	“I don’t know of large outcome trials to look at long term fetal effects meaning risks of things like growth restriction, long term neurologic development, etc. on drugs like sofosbuvir, etc.” (MFM)
	Patient hesitancy to treat during pregnancy	“I think for a lot of pregnant women in general whether it’s hepatitis C or another disease or anything, a lot of women are really nervous about treatment of any kind in pregnancy because they’re usually really concerned about what that would mean for their baby” (MFM)
Health inequities and access concerns	Implicit bias and stigma	“I mean I think in terms of that [adherence] I think providers should be judgmental….I mean I don’t think you know providers should be judgmental and say well, this person is not gonna comply so I’m not gonna give her this medication. But it’s gotta be done safely, you know?” (Ob/Gyn)
	Access and cost	“…I have a huge practice that has Medicaid because [Midwest state I work in] gives Medicaid to poor people who are pregnant, but as soon as they’re no longer pregnant, they no longer have insurance. So I could frequently be in a position where the patient may be able to afford something when pregnant but would lose the ability to pay for it after her delivery. So we talk about that when offering things.” (Ob/Gyn)
	Unfamiliar with local HCV burden due to no screening	“At the time in the population that I saw not very common at all. I think we also weren’t routinely screening for it. Certainly not in pregnancy and I’m not sure when it became part of a general STI screen, but I don’t know that I really ever saw it as a resident.” (Ob/Gyn)
Anticipated adherence and engagement challenges	Treatment as an added pregnancy burden	“Well, in terms of if there’s a treatment what they can do, like how does it work for her not just finances but also transportation and childcare, those sorts of things in terms of making something accessible to a patient” (Ob/Gyn)
	Perceived limited health literacy	“I think it really depends on the patient. I think that there’s a wide range of health literacy among patients. There are patients with diagnoses who actually know more about their diagnosis than we do, particularly if they’ve had it for a while and are well educated but there are also patients with poor health literacy who don’t even have a good understanding of what it is. I think it’s a wide spectrum.” (MFM)

First, regarding insufficient knowledge, obstetricians identified subthemes of *lack of training*, *misconceptions and incorrect knowledge*, and *infrequent exposure to patients with HCV*. One Ob/Gyn noted, “I mean I would say I try to keep up to date on ACOG bulletins and things like that about it, but formal training [on newer treatments for HCV], no.” Another stated that when talking to patients, they felt, “I would be honest about the fact that I…don’t have a lot of experience with this and I would need to look things up and I would.” Others had incorrect knowledge about HCV treatment, such as not appreciating the advances with DAA therapy and the potential benefits that pregnant individuals may experience (e.g. possibility of aligning HCV treatment monitoring with antenatal care or the possibility that cure during pregnancy may eliminate the risk of perinatal transmission). Furthermore, clinicians identified that their infrequent exposure to HCV diminished their familiarity with treatment options. One MFM stated, “…I have a much less broad understanding of the side effects of the medications used to treat hepatitis because I just don’t have a lot of experience with administering them.”

Second, obstetricians identified an inadequate evidence base and uncertainty about safety as major barriers. The subtheme of *lack of professional guidelines* predominated, with both MFMs and Ob/Gyns identifying the lack of guidelines by ACOG or others as a major deterrent to initiating treatment. A MFM stated, “I use the societal guidelines of my society, of ACOG, and I treat in pregnancy like hepatitis B, HIV. Hep C is the one that right now there’s no guidance on treating in pregnancy.” Similarly, an Ob/Gyn stated, “I would certainly want to get the most current guidelines.” Others noted that it was actively recommended to not treat, such as the MFM who stated “right now it’s not recommended to treat hepatitis C in pregnancy.” Obstetricians also generated the subtheme of *lack of pregnancy-specific safety data* as a related barrier. A MFM stated, “[We need to m]ake sure that there’s no, we know that some medications increase risk for pregnancy complications such as preeclampsia or prematurity” and another similarly stated, “[We need data on] fetal long-term outcomes as well as evaluation of fetal growth…I don’t think we have robust enough numbers to say that this does not [harm] and really long-term neurologic outcome for these kids with exposure to this medication is a big one.” Additionally, obstetricians felt patients would have safety concerns, noting *patient hesitancy to treat during pregnancy*. When describing general safety concerns about medication reluctance during pregnancy, an Ob/Gyn stated: “I imagine that there is probably a subset who is very hesitant…because many of my patients are very hesitant about medication use in pregnancy.” This hesitancy was described in the context of the lack of HCV-specific evidence: “I think patients are more hesitant to accept newer medications because they think that they’re not as well studied or we don’t have long-term data on fetal outcomes.”

Third, obstetricians identified health inequities and access concerns as barriers to treatment during pregnancy. *Implicit bias and stigma* towards patients with HCV were considered to be health equity concerns (Table 2), although the most prominent subthemes were regarding *access and cost*. The majority of obstetricians had concerns about the cost of HCV therapy, noting that both publicly- and privately-insured patients had limited access to DAA therapy. One MFM stated:

“I know that in [Midwest state where I did my fellowship] it was difficult to get patients covered, primarily because a lot of our patients who had hepatitis C had a history of IV drug use because we had a large opiate population. So many of them were on Medicaid in which case their Medicaid coverage ended after six weeks postpartum, so that was a huge issue…So I do not know [as much about Illinois] but I assume it [access] is poor.”

Similarly, MFMs who had familiarity with HIV treatment during pregnancy noted that access to DAA therapy is considerably more challenging than access to antiretroviral treatment, citing HIV-specific drug access programs: “Unlike ADAP which ensures coverage of all HIV-infected patients to the therapy that’s necessary for the treatment of their disease, the same guarantees do not exist for Hepatitis C.” Access to specialists with expertise in HCV treatment was a related barrier. A third subtheme related to access was that obstetricians were *unfamiliar with the local HCV burden due to no screening*; clinicians identified that their lack of knowledge regarding HCV diagnoses likely prohibited equitable access.

Fourth, obstetricians described their anticipated patient adherence and engagement challenges as barriers to treatment. Subthemes included *treatment as an added pregnancy burden* and *perceived limited health literacy*. A MFM described their concerns about non-adherence and the burdens of treatment:

“We know that non-adherence to medical care has its own set of comorbidities associated with it and if we had a medication that potentially had fetal harm that a patient wasn’t going to adhere to and not receive the benefit of doing the medication, there’s a question as to whether or not we should be giving it then. So I think that’s where it would come into play and the discussion that I would have with the patient if they didn’t feel like they could be compliant with the treatment and it had a potential risk, then I wouldn’t encourage them to do the treatment.”

Another described the role of social determinants of health: “[S]ome of it is health education and health knowledge, some of it is the structural and social determinants like what else is going on in their lives that enables them to engage in any health behavior.” Another described the schedule of taking medications:

“The patient has to be engaged, right? So obviously there are some medications that require a more rigorous schedule, following up more frequently. So you know I would prefer someone who was really involved in their care, was an advocate for themselves and they themselves had a vested interest in the treatment.”

Obstetricians’ perceptions of the burdens of treatment and limited health literacy were seen as factors that would diminish their likelihood of recommending treatment, because they were concerned patients would not be sufficiently adherent.

### Patient-identified barriers (Table 3)

Patient participants identified six overaching themes regarding barriers to HCV treatment during pregnancy, including: insufficient provider knowledge, provider communication and process challenges, access and cost, psychological barriers, safety concerns, and unclear rationale for treatment during pregnancy. Although some patients had previous experience with older therapies, analyses regarding treatment experience or expectations were focused on DAA therapy.

**Table 3:** Patient perceptions of barriers to treatment of HCV during pregnancy.

Theme	Subtheme	Exemplary quotation*
Insufficient provider knowledge		“[I would] probably [rate how I changed my behavior as] a 10. I have listened to everything that my doctor has told me in this pregnancy. I’m seeing a different doctor than I did the last time just because I don’t think they had known enough information about having hepatitis while pregnant because it didn’t seem like they were very confident in the information they had given me, so it’s definitely different this time with the doctor I’m seeing now so.”
Provider communication and process challenges	Provider communication barriers	“Since my doctor is telling me that it was too risky and could be too harmful, so I pretty much decided not to do it and took his advice but like not being able to really find much info about it, I guess the doctor’s opinion really like makes a huge difference in probably a lot of people’s decisions because people trust their doctors.”
	Perceived lack of provider compassion	“You gotta care, you gotta care about your patient, you gotta care about the child that patient is carrying, you gotta care about the patient and the child that patient is carrying more than you care about fixing a disease.”
Access and cost	Insurance limitations	“So to be honest I just kinda gave up [due to insurance hurdles]… then the next doctor that I saw, he said hey, let’s try this and even if we get denied I’m gonna appeal it until someone offers you new medication. So I actually did not too long ago.”
	Cost	“I had a few situations as to where I may not have been able to finish treatment because the insurance would not pay for it and they were like, so of course your question would be well how much is it? Maybe I can pay for it because you’re thinking just a couple hundred dollars but you know you’re able to get the hundred dollars together because you’re like oh this is for my health, I’ll pay for it. So it was like thousands and they were like oh this treatment, it’s like $10,000 a month or you know I was just guessing. I don’t really recall but it was a lot, it was a lot and I was just like oh well, so much for that.”
Psychological barriers	Feeling overwhelmed by HCV diagnosis and treatment	“The emotional support really needs to be provided for and offered and opportunities for that. Instead of here, just see this doctor, just see this doctor, oh we have this support group for you, here we have. You know like that would be a thousand times more helpful to all these ladies than you know because I think if a doctor is interested in treating a woman while she’s pregnant, chances are good, then she just found out she has hepatitis C and is on oh my gosh, what am I gonna do, how am I gonna take care of this kid now? They’re not even born yet and now I’ve got this death sentence and oh my gosh, what am I gonna do? Oh you’re telling me I gotta do this right now?”
	Stigma	“I’d say it’s a pretty big deal. My daughter had gotten it, even though I tried to, I’m sorry, I don’t know why I’m getting emotional…My daughter got it, even though I tried to you know take care of myself so she would be healthy.”
Safety concerns	General concerns about medication use	“So they’ll be like oh it’s okay, go ahead and take it but now with more modern medicine…I don’t know what they put in this, I don’t know if there’s something that’s in this that can hurt the baby, so 9 times out of 10 unless it was just like I gotta chance it, I just didn’t take anything.”
	Side effects	“I had heard the side effects from it were kind of harsh. So I think that deterred me from even wanting it, ya know? I wasn’t very interested after hearing that because I was scared of what could happen”
	Fetal safety	“They told me while I was pregnant I could not have treatment…You know I understand like when you’re pregnant you couldn’t do the treatment. Obviously, you know because of the baby or whatever and risks because of that.”
	Lactation	“What kind of role would it play for me as far as breastfeeding? Would that cause me not to be able to breastfeed? Would that be something that could go through my breastmilk because if I’m taking it and I’m not finished with it when my baby is born, you know is it something that’s gonna harm him or her through breastmilk?”
Unclear rationale for treatment during pregnancy	Precautionary principle	“I definitely thought about the pros and the cons and the risks if I was to not follow the medical advice. So I guess prioritizing whether or not it was something that needed to be addressed during pregnancy or if it could wait until afterwards.”
	Opposition to treatment regardless of evidence	“I’m pregnant, there ain’t no way in hell I’m going anywhere near anything [treatment] while I’m pregnant. I think it’s very selfish for a parent to want to treat themselves for something that they have while they are pregnant. I also think that it’s very selfish for doctors to want to treat patients when they’re pregnant because there is no way, no reason that that can’t wait nine months. Your body is already under extra taxation when you’re pregnant, ya know? Like in addition to you’re tending to someone else. Like the concept that someone even would consider being treated for hepatitis, I mean I haven’t been treated in almost 20 years since I found out and the concept that somebody can’t wait nine months is absolutely absurd in my mind both from a patient perspective and from a medical provider perspective. I don’t think that they should even offer treatment when someone is in term.”

First, regarding insufficient provider knowledge, patients identified a wide range of clinician experiences, including many experiences in which clinicians seemed to have little or no knowledge about HCV treatment. One patient, who had received no HCV treatment to date at the time of the interview, stated:

“I didn’t learn much about treatment options because the general practitioner, the internist that I saw at the time, he didn’t know much about what the treatment options were…He did say that there’s treatment available. He did also say that at that time it was very time intensive, a very difficult treatment to undergo but not much.”

A patient who was treated after her pregnancies identified a similar problem with her obstetrician:

“[O]ne thing I wish would have happened somewhere along the way was that I would have been given treatment options. You know I don’t blame my OB. I know that’s not his…area of expertise but I believe the treatment has been out for some time before it was offered to me, so I just look at like how easy the treatment was, how well I reacted to it and that it wasn’t offered to me sooner.”

Second, the theme of provider communication and process challenges encompassed two subthemes: *provider communication barriers* and *perceived lack of provider compassion*. Individuals who identified communication barriers noted the lack of provision of information as well as a lack of hope. This theme was distinct from the lack of knowledge, focusing instead on the manner in which a provider communicated, or failed to do so, rather than on insufficient knowledge or experience. For example, one participant stated, “Back in 2016 I saw a GI doctor, he gave me no information and no hope and then I saw [community health center] in 2018 and that’s when…she was a specialist, so she gave me a lot of hope.” Clinicians perceived to lack compassion were described by some patient participants when recounting their experiences of seeking care. For example, a participant described a conversation with her physician in which she requested information about treatment, stating: “He pretty much shut me down immediately when I had talked to him about it.”

Third, access and cost were major patient barriers. Nearly every patient participant described experiencing either *insurance limitations* and/or *cost* as obstacles to HCV treatment. Participants described complex and burdensome experiences with seeking treatment coverage, as well as costs associated with treatment, such as for the costs of appointments and surveillance. One patient stated:

“I did see a specialist for treatment after I had my daughter, but the cost of the treatment was so substantial that I just didn’t even look into it after that. You know it was a lot…The only time that I ever tried [to get treatment]…I just remember my insurance not covering very much of it and that it was really expensive and that’s what stopped me from even getting it, because there was no way I was gonna be able to even afford like a quarter of the cost.”

Fourth, within the theme of psychological barriers, two subthemes emerged: *stigma* and *feeling overwhelmed by HCV diagnosis and treatment*. Stigma associated with HCV was seen as a psychological barrier to initiating care, particularly when their children had acquired HCV via perinatal transmission. One participant described the need for trauma-informed HCV care, stating clinicians need to:

“[T]rickle in some more trauma therapists in general because many people have hep C due to either a series of traumatic events or some sort of trauma related to how they got it…I just think just to add more, like trauma specific either group or therapy where you really just kind of dig into the story of how they got hep C rather than just maintenance.”

The feeling of being overwhelmed was a limitation to treatment engagement, such as the participant who stated, “I was actually at work when I got a phone call and…any time that you get any kind of information…health related you almost feel like it’s a death stamp…you’re not really thinking when you get your diagnosis because you’re just being bombarded in your own brain.”

Fifth, patient participants identified multiple safety concerns. Safety subthemes included *general concerns related to medication use* during pregnancy, *side effects* specific to HCV treatment, questions about *fetal safety*, and concerns about *lactation*. Several participants noted a general preference for non-intervention during pregnancy, noting that their perspectives on HCV treatment did not differ significantly from concerns about other medication use during pregnancy. Others described more specific pregnancy or lactation concerns, including some who were told to avoid treatment due to safety concerns. One participant stated: “I was actually talking to the doctors in both pregnancies, but they did not recommend to start the treatment when I was pregnant. I was waiting until I finished breastfeeding my second kid to start the treatment because they did not recommend treatment during the breastfeeding as well.”

Finally, patients discussed the concept of an unclear rationale for treatment during pregnancy versus delaying treatment until after delivery (Table 3). Some patients described the *precautionary principle*, in which they were deliberately weighing the risks and benefits and potentially having a risk tolerance level that favored postpartum treatment rather than treatment during pregnancy. Most patients opposed to treatment were in this group, in which they generally demonstrated a willingness to consider treatment based on the evidence, but felt the current state of evidence favored waiting. A minority of patients demonstrated the subtheme of *opposition to treatment regardless of evidence*, in which they described that treatment during pregnancy would never be an option regardless of clinician recommendation or level of evidence.

### Patient-obstetrician barrier concordance and discordance (Fig 1)

Patients and obstetricians perceived several similar barriers, particularly issues of cost and access, insufficient provider knowledge, and uncertainty about safety. However, we identified areas of discordance. Obstetricians rarely recognized stigma and the psychological barriers identified by patients. Additionally, obstetricians identified health literacy as a concern that may limit their offer of treatment to patients, although patients did not describe their own health literacy as a barrier to HCV treatment. Patients similarly did not endorse the obstetrician concerns that adherence challenges and logistical burdens (daily treatment, need for monitoring, and role of social determinants on adherence) would preclude successful treatment during pregnancy. One patient noted that although taking a daily pill can be hard, she did not see this as an obstacle: “[My experience with treatment] was fine. Just taking one pill again once a day. It was just trying to remember the same time every single day was a little bit harder, so if I did forget I wrote the nurse and was able to get that taken care of.”

**Fig 1:**
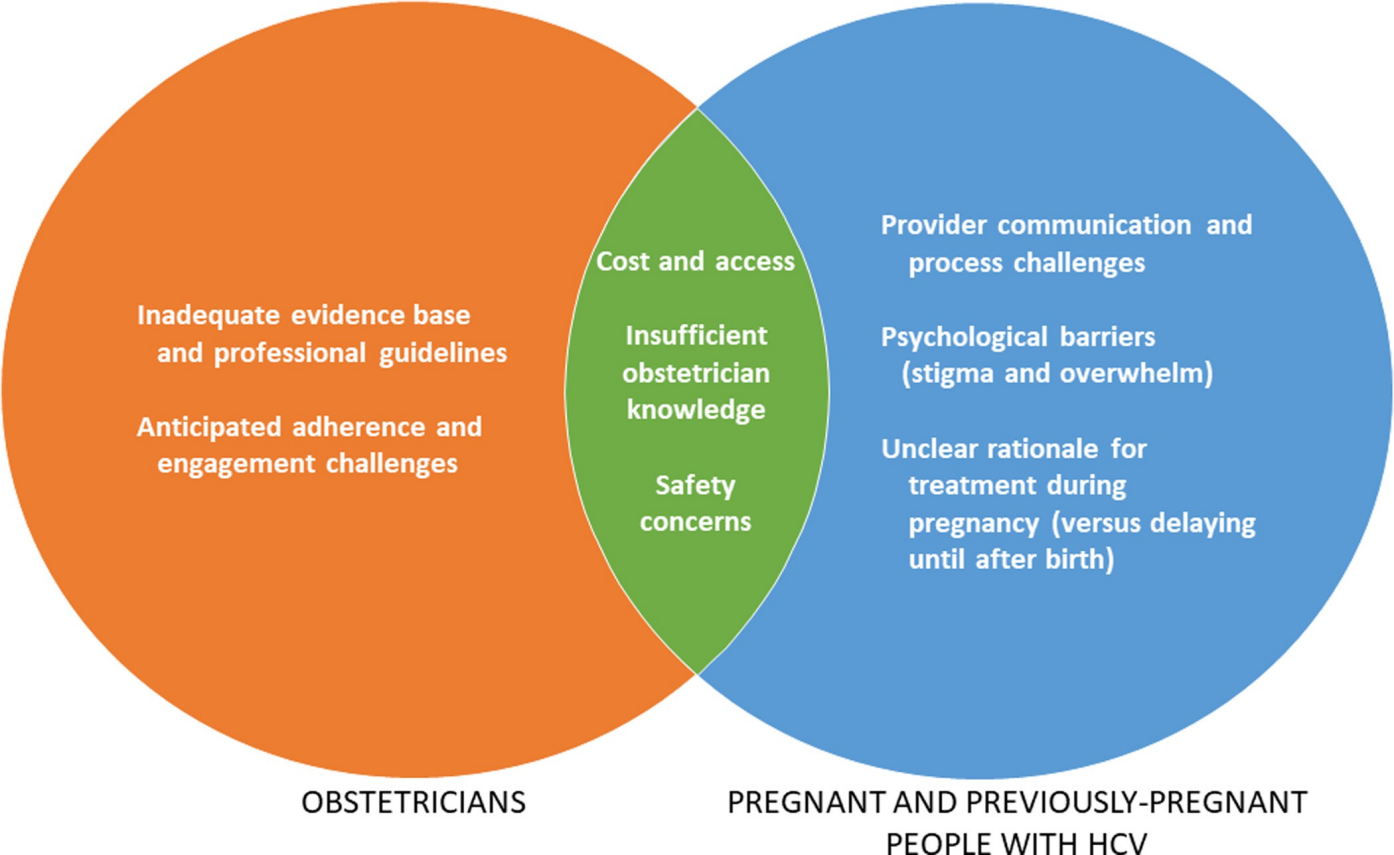
Patient and obstetrician-identified barriers to inclusion of pregnant patients in HCV treatment programs.

### Patient and obstetrician-perceived facilitators (Table 4)

In contrast to the discordance identified in multiple barriers, patients and obstetricians were more aligned with regard to facilitators of treatment. Facilitator themes included: pregnancy as a window of opportunity for treatment, interest in maternal health benefits, and prevention of perinatal transmission. With the exception of the healthcare access subtheme, which was only described by obstetricians, all other themes were generated by both patients and obstetricians.

**Table 4:** Patient and obstetrician-perceived facilitators of treatment of HCV during pregnancy.

Theme	Subtheme	Patient example	Provider example
Pregnancy as window of opportunity for treatment	No reason to defer	“I think as long as the risk is, the pros and cons, as long as the pros outweigh the cons it is worth a try…if you are given the chance I don’t believe that you should have to wait and as long as it’s safe for the mother and baby, then yes, go ahead and continue your treatment or start a treatment while pregnant.”	“I think it’s a good idea. I mean it’s something that I think should be explored more. I think we need more data to say that there’s long-term safety, but I think it is very reasonable. We get a lot of patients tuned up on their other comorbid conditions during pregnancy because of that you know window of opportunity so I think it’s a good idea.” (MFM)
	Enhanced health care access during pregnancy	(no comments)	“There’s a lot of women who don’t have insurance and they only have insurance during pregnancy and six weeks postpartum. Hopefully we’re changing that for a year postpartum which would be fabulous. So again pregnancy is a window of opportunity to get patients treatment. They may not be able to you know once their pregnancy is over.” (MFM)
	Enhanced motivation for behavior change during pregnancy	“I just decided, I just wanted to do everything that the doctors had told me down to a T just to make sure that everything that happens with the baby and the decisions I make for the baby are for the better of the baby…I have asked also if there’s treatment during pregnancy but there’s not. but you know they do give you information and different things that you can do that can help you, like not smoking cigarettes…so I’ve definitely steered cleared of those things.”	“I mean pregnancy motivates people to do all sorts of stuff. It’s one of those times that people are able to make significant lifestyle changes for the sake of their baby. I mean there’s just this whole big other motivating factor there…I think people are very much interested in trying to be as healthy during their pregnancy for both themselves and their baby.” (Gen Ob/Gyn)
Interest in maternal health benefits		“I know my like conscience is now clear that I’m not gonna infect anyone. That was my main concern about when I had hepatitis C….Then second of all when I cleared I knew that my liver is finally gonna work sort of normally because now when I was hepatitis C it was working too much, and it was like a rock on it and it was working too much and now she’s clear, she doesn’t have the virus inside…now I feel like my liver works easy.”	“Treating hepatitis C obviously is important for maternal health. (Gen Ob/Gyn)
Prevention of perinatal transmission		“Then now like I wouldn’t have to decide or have to consider like be worried about passing it to my baby…and I was always having to be careful, afraid of like infecting somebody else so it’s not, it’s just more not a constant worry is the biggest benefit [of treatment]…Like even over my health and my liver function and all of that, it really helped my mental stability a lot more.”	“I would imagine if there is risk of vertical transmission the hope would be the treatment in pregnancy could reduce that risk. I would also hope that if there was concern about progressive liver disease that treatment in pregnancy could prevent that from progressing.” (MFM)

Regarding pregnancy as a window of opportunity, three subthemes emerged: *no reason to defer*, *enhanced healthcare access*, and *enhanced motivation for behavior change*. Patients commonly reported that if there was no harm to treatment, they saw *no reason to defer*. One person stated, “If it was during my pregnancy, as long as it was safe I’d do it for the baby” and another described, “I had to wait until I was done with my pregnancy. If there’s no, you know if it’s not harmful to the baby or anything or you know doesn’t affect him in any way, I think it’d be great.” Similarly, a MFM described pregnancy as a time to optimize health:

“I think it’s a fantastic window. I think that is in general for young women that is often the prime opportunity to get women care in all sorts of medical specialties because it’s really the only time that they see a doctor. When they’re young if they’re healthy otherwise or have no perceived acute health care needs, that’s our window of opportunity to screen them and get them into care.”

Patients noted that it would not be a “big deal” to take medication during pregnancy, stating “You just take the medication and it’s noninvasive and as long as you take it every day you should be clear…It just made me feel reassured.”

Pregnancy was also seen as a window of opportunity because of *enhanced health care access during pregnancy*. In many states, including Illinois, low-income pregnant individuals have access to health insurance that may not exist outside of pregnancy. One MFM stated, “I also believe a primary reason why people are interested in treating during pregnancy because it’s a small window when people actually have good health insurance.” Additionally, beyond insurance, greater interactions with the healthcare system during pregnancy was seen as a facilitator of the DAA treatment regimen.

Although patients did not describe the healthcare access subtheme, they concurred with obstetricians that pregnancy is a period of *enhanced motivation for behavior change*. This sentiment was echoed by multiple patients regarding their general health and HCV-specific health, as in the patient who stated: “When I found out I was pregnant I was homeless and a drug addict and an alcoholic…I immediately stopped, went in for a prenatal care and have been sober ever since…I’ve been eating regularly and sleeping as much regularly as I can.…Completely different.” Another participant stated she would rate how she changed her behavior during pregnancy as “a 10; I have listened to everything that my doctor has told me in this pregnancy.” Others similarly described their motivation for lifestyle changes and willingness to engage in clinician recommendations during pregnancy, such as the participant who stated “I had to change all my eating habits, do a lot more mediation…I did overall take their [doctor’s] advice because it was my first child and his life is more important than mine.” An Ob/Gyn agreed:

“I think pregnancy is a big motivating factor for women in terms of improving their own health just for the sake of they will have a child that they have to take care of and also in terms of them not wanting to do anything to harm the fetus during the pregnancy or the child afterwards. That is often a big motivating factor.”

Second, interest in maternal health benefits was a facilitator for both participant types. One patient stated she would be willing to accept treatment because:

“if it means that you might get sicker and then not be around for the baby then I’d say take it. Like it just depends on the risks and you know the viral level and where they were in treatment before they got pregnant. I’d be open to [taking HCV treatment during pregnancy] if it means that it’s the best for everybody.”

Another stated, “I heard there’s a 98% cure rate with my type of hepatitis C and the medicine they’re going to prescribe me.” Similarly, a MFM stated, “I think I would consider treating for maternal benefit alone even if there was no risk of vertical transmission or low risk of vertical transmission, just for maternal benefit.”

Finally, prevention of perinatal transmission was motivating for both patients and obstetricians. Multiple patients underscored that prevention of perinatal transmission would be a major incentive to initiate treatment. One stated, “I think if it’s safe or… a doctor is recommending it and is weighing the pros and cons and if the pros outweigh the cons, I think it’s something that somebody should do for both their selves and their child’s health. I think that if it minimizes the risk of your child contracting it, then it’s well worth it.” Obstetricians echoed that a goal of treating during pregnancy is preventing perinatal transmission: “We begin to treat her disease, hopefully rendering it in remission and potentially reduction in perinatal transmission.”

## Discussion

While the goal of eradication of HCV is within reach given the curative power of DAAs, exclusion of pregnant individuals from HCV treatment programs could frustrate the achievement of this goal. Furthermore, limiting the potential benefits from HCV treatment for pregnant persons and their children raises justice concerns. Patient and clinician perspectives are key to determining the appropriate way to include pregnant individuals in HCV treatment programs. We identified several patient and obstetrician barriers and facilitators to inclusion of pregnant individuals in HCV treatment programs. Both groups were generally receptive to HCV treatment in pregnancy and recognized pregnancy as an important window of opportunity for treatment. Concerns about cost, access, and equity were shared by clinicians and patients. Obstetricians did not identify patient concerns about stigma, however, and patients reported their clinicians did not always counsel them appropriately. Furthermore, we identified obstetrician knowledge gaps, and only obstetricians were concerned about adherence and engagement challenges among patients with low health literacy.

Given widespread adoption of universal screening for HCV among US adults, leading to increased HCV screening in obstetric practice, [9–11] important next steps include developing programs that include pregnant individuals in treatment programs when HCV is identified. There are many reasons that pregnancy provides a crucial window of opportunity for HCV treatment [22]. As obstetricians identified, pregnancy is a time period in which low-income individuals may access health insurance that may not otherwise be available. Pregnant individuals have enhanced interactions with the healthcare system, including frequent prenatal appointments that include laboratory surveillance. HCV treatment is ideally suited to integrated into other obstetric health services such as antenatal social services programs. While further data on safety are needed, the 8 to 12 week duration of DAA therapy means therapy may be initiated after organogenesis is completed and conclude before the individuals gives birth. Finally, treatment offers potential benefits for pregnant people and their infants. HCV treatment may reduce or eliminate the risk of perinatal transmission and other clinical sequelae of HCV during pregnancy [22]. However, further research to establish the evidence base for the safety of HCV treatment during pregnancy is essential to support the development of strong recommendations for treatment, which are viewed by participants in this study as critical missing facilitators.

Relatively little work addresses patient and clinician perspectives on treatment of HCV during pregnancy. Kushner et al surveyed patients and identified a high willingness to take DAA therapy during pregnancy if it would result in prevention of perinatal transmission; factors associated with receptivity to treatment included being of reproductive age range and having a prior history of HCV treatment [24]. These attitudes were echoed by pregnant participants in a phase 1 DAA trial [23]. In qualitative work exploring DAA implementation in the primary care setting, prescribing providers in Delaware expressed positive attitudes regarding HCV treatment, while also identifying patient- and practice-level barriers [30]. As in our findings, these barriers included limited preparedness to treat, infrequent exposure to HCV, and concerns about psychosocial challenges [30].

To address gaps in obstetrician knowledge that serve as impediments to HCV treatment initiation, prior work in the primary care setting suggests that key next steps may include enhanced clinician education and dissemination of information about the current state of evidence [30]. Better coordination between infectious disease, gasteroenterology, and obstetric experts may facilitate such knowledge transfer. Additionally, educational efforts that interface with public health and community agencies and include organizational support may be key, as siloed efforts are unlikely to yield widespread information dissemination and practice change [30]. Joint public health-community-academic efforts to promote knowledge about HCV treatment during pregnancy may be modeled after successful partnerships designed to eliminate perinatal HIV transmission, such as those designed to support the Illinois Perinatal HIV Prevention Act [31–33].

Patients also identified psychological burdens and stigma as barriers to HCV treatment during pregnancy, although obstetricians did not identify these concerns. Stigma reduction efforts and trauma-informed obstetric care [34] for people with HCV must be undertaken in order for patients to fully engage in and benefit from HCV treatment programs. In addition, we identified an undercurrent of medical paternalism in which obstetricians were concerned about adherence or acceptability due to their perceptions of the burdens of treatment, the role of social determinants of health, and perceived limited patient health literacy. Yet, patients largely reported that with compassionate, patient-centered, inclusive, and empowering health care, they would be desirous of treatment and would not perceive the logistics of treatment to be major burdens. Our findings speak to the importance of centering patient voices and underscore the importance of person-centered decision-making [11].

Despite the identification of multiple barriers, both obstetricians and patients identified pregnancy as an important window of opportunity for treatment. Both groups also recognized that the achievement of HCV cure during pregnancy would provide lifelong maternal health benefits and potentially prevent perinatal transmission. This positive perspective has been noted in other studies [24, 25]. In work by Kislovskiy et al, interviews of nine pregnant people who were enrolled in an open-label, phase 1 study of ledipasvir/sofosbuvir during pregnancy yielded themes related to treatment during pregnancy being tolerable and convenient; taking DAAs increased individuals’ sense of well-being due to the possibility of cure; and participants appreciated the provision of non-judgmental, person-centered care and communication [23]. Although Kislovskiy et al.’s study was conducted among individuals who had already agreed to enroll in an early stage trial, it is reassuring that these individuals similarly identified a cure from HCV as “life changing” and were very positive about antepartum treatment [23]. In addition to these specific motivations for HCV treatment, pregnancy represents a pivotal opportunity for treatment due to the unique health care access experienced by pregnant individuals, some of whom may not have insurance or regular contact with a clinician outside of pregnancy. Recognizing these powerful motivations and opportunities for treatment in pregnancy may be essential to eradicating HCV.

Both groups raised cost of treatment as a barrier, but obstetricians also expressed the opinion that their knowledge of HCV was not up to date and some patients received their HCV treatment several years ago. When they were first introduced, DAAs were priced exceptionally high (>$150,000 per course of treatment) and the specific pharmaceutical companies received considerable negative media attention as a result [35–37]. In recent years due to competition and fixed-cost access programs, the cost of therapy has come down considerably (~$25,000 or less per course of treatment) [18, 19, 38]. Our interview guide did not explore details about how the current cost of therapy may influence perspectives, but it is interesting to consider how specific cost estimates may impact the attitudes of both patients and clinicians.

Our findings suggest a number of future directions regarding clinical, public health, and educational programming to reduce barriers and promote HCV treatment. From a clinical intervention standpoint, potential evidence-based interventions to promote patient participation in HCV treatment programs include the use of patient navigators or enhanced case managers who can address social determinants of health that may be barriers to participation [39, 40]. Such care coordination efforts may include managing treatment during pregnancy or, if preferred, the management of immediate postpartum treatment processes via referrals to specialist care and coordination of insurance approvals. Other care coordination efforts may include centralized referral services, such as public health “warmlines” that mirror the work being conducted with perinatal HIV hotlines [32, 41]. Additional clinical interventions may include the development of decision aids to support knowledge acquisition and shared decision making processes. Further, with increased education of obstetric providers, HCV treatment programs could occur within the context of routine antenatal care, rather than via the construction of distinct programs that further fragment care. From a public health and research perspective, there is a compelling and urgent need for greater research regarding HCV treatment during pregnancy, as the lack of evidence remains a major barrier to acceptance by obstetricians and patients [22]. The limited federal support for such research programs has led to slow development of new knowledge about HCV treatment safety in pregnancy. Public health efforts must also include policy efforts to include insurance coverage for HCV treatment during pregnancy. Additionally, advocacy and educational efforts are essential to reducing stigma, highlighting the importance of treatment, and addressing potential patient concerns about participating in treatment and research programs. Future work must study the role of educational or decision support interventions. Academic-community partnerships, including focusing on community-based advocacy organizations and their grassroots expertise, may also be an important strategy to improving treatment acceptability. Future work may also consider expanding investigation to infectious disease and hepatology specialists to better understand their perspectives on the findings identified herein.

Strengths to this analysis include that it was an in-depth, rigorous investigation of attitudes about HCV treatment in pregnancy conducted among diverse stakeholders. As one of few such investigations, it provides valuable information on how best to promote HCV therapy during pregnancy. However, several limitations warrant consideration. As is typical for qualitative research, the findings are not intended to be generalizable. Participants were patients of, or clinicians affiliated with, a large, academic medical center and its community affiliates, which may bias them towards greater knowledge and access. Selection bias may exist by the inclusion of individuals with up-to-date contact information who were identified through the health care system. In addition, obstetrician participants were largely female (as is typical for this field) and practicing in an urban setting. Further research is required to understand the perspectives of other diverse clinicians. Finally, our study period aligned, by chance, with a practice change recommendation by the USPSTF (released March 2, 2020), but our study design did not allow for the examination of differences in attitudes based on the change in screening recommendations.

In conclusion, HCV treatment during pregnancy is perceived by both obstetricians and patients to have potential clinical and public health benefits. Both obstetricians and patients identified barriers to the inclusion of pregnant individuals in HCV treatment programs, including issues with cost, access, safety, and knowledge. Overcoming concerns about safety represents an important next step, highlighting the need for high-quality data regarding the safety of HCV treatment during pregnancy. However, patients largely recognized that barriers may be mitigated with greater knowledge and compassionate, person-centered care. These informational and access barriers must be addressed to ensure the responsible inclusion in HCV treatment programs for pregnant individuals.

## Supporting information

S1 Appendix Provider and patient interview guides (Version 1.0). (DOCX)
