# Supplementary material for: Identifying barriers and facilitators of the inclusion of pregnant individuals in hepatitis C treatment programs in the United States
Source: PLoS One. 2022 Nov 18;17(11):e0277987. doi: 10.1371/journal.pone.0277987 (PMC9674123; doi:10.1371/journal.pone.0277987)
Supplement: S1 Appendix — (DOCX) [file pone.0277987.s001.docx]

**Appendix – Provider and Patient Interview Guides**

**Version 1.0**

**OBSTETRIC PROVIDERS**

**Introduction** *(consent to have been done in advance)*

Thank you for joining us today. You have been asked to participate in this interview because you are a physician or midwife who has cared for women with hepatitis C.

In order to expand our knowledge of how to best care for women with hepatitis C, we would like your feedback regarding your knowledge and experiences with patients like this. I will ask you some questions on your experiences, your familiarity with hepatitis C and its treatment, and your opinions on these issues as related to pregnancy.

Before we begin, I just want to remind you that there are no wrong answers. We are only trying to get a better understanding on how to best care for pregnant women with hepatitis C. We welcome all perspectives and we very much appreciate your time and willingness to share your expertise.

The information you share today will remain completely confidential. Everything you say will be audio recorded, transcribed, and stored on a password-protected computer. Please avoid using your name or other identifying information in this interview – if your name or other identifying information is mentioned during the interview, we will not use it in reports. You can choose to participate and to stop participating at any time. Do you have any questions?

**Part 1: Clinical background and experiences**

- Tell us a bit about yourself.
  - What is your role within Northwestern Medicine or the organization you work for?
  - Tell us how long have you been working in this position?
  - When did you finish your final clinical training (i.e. residency, fellowship, or CNM training)?
  - Where in the country did you complete your training?
    - Was it urban or rural?
    - Did your training site(s) have a reproductive infectious disease fellowship?
  - How common was Hep C in the geographic area where you trained?
- Tell us about your current patient population.
  - How many total pregnant patients do you see annually?
  - How many patients with Hep C have you seen in the last year?
  - If you had a pregnant patient with Hep C in your practice, how would you manage her?
    - Would you keep her in your practice or recommend care elsewhere?
- Have you ever had any training or reviewed any literature about newer options for treating hepatitis C?
  - If you were trained, where did you have that training?
  - Have you consulted or are you aware of any guidance on Hep C treatment in pregnancy?
    - *[Potential probes]: American College of OB GYN (ACOG)/Society for Maternal Fetal Medicine (SMFM), American Association for the Study of Liver Diseases (AASLD)*

**Part 2:**

We’d like to hear your opinions about balancing maternal and fetal interests.

- How do you make decisions about treatment when evaluating an infectious disease during pregnancy? What factors do you consider?
  - Tell us about how your decision making process changes with…
    - …a chronic infection?
    - …an infection with risk of fetal transmission?
      - *[Probe If provider brings up Hep B]: Do you think there is a difference between treating Hep B versus Hep C during pregnancy? **Background info for interviewer to be aware of: Hep B’s goal is to prevent transmission to fetus, Hep C’s goal is to treat mother and fetus***
    - …non-vertical transmission fetal morbidity?
  - How does patient engagement and ability to adhere to therapy alter your recommendations?
  - How does insurance coverage alter your recommendations?
  - What other patient factors do you consider – for example, health literacy, co-infections, substance use, social support, or other factors?
  - Consider HIV management during pregnancy. When you think about how we manage HIV during pregnancy, how is it similar to or different than Hep C?
    - What do you think about the different goals?
      - The goals of HIV therapy are viral suppression, which is the mainstay of strategies for preventing fetal transmission and prevention of OIs, whereas the goal of Hep C therapy is virologic cure, which would then theoretically prevent fetal transmission.
    - What do you think about the potential risks of HIV treatment v. Hep C treatment?
    - What do you think about the potential benefits of HIV v. Hep C treatment?

Next we’d like to talk about your knowledge and opinions regarding viral hepatitis in pregnancy. In this section we want to understand what providers know about Hep C treatment. We’re looking for providers with all levels of knowledge and experience with Hep C treatment, we are not trying to quiz you, and this interview will be anonymous and none of your answers will be linked to your name.

- Who do you think is at risk of Hep C?
- Tell us about your knowledge of untreated Hep C in pregnancy.
  - What are the maternal risks of Hep C?
  - What are the fetal/perinatal risks of Hep C?
  - What is the risk of perinatal transmission of Hep C?
  - How would you alter prenatal care or intrapartum management for women with Hep C?
  - What are the risks of co-infection with HIV or other infections?
- Tell us your understanding of the nature and outcomes for current Hep C therapy.
  - How is it administered?
  - How often would one take the treatment?
  - How long does treatment take?
  - How well does it work?
  - What are the risks of treatment?
  - What are the names of the treatments?
- Are you aware of the current cost range for the therapies and payment options?
- How available is treatment at your institution, region, or state, for patients in general?
- Tell us about your knowledge of treatment in pregnancy.
  - What is the current data for treatment in pregnancy?
  - What are the potential benefits and harms of treatment of Hep C during pregnancy?
- What other things related to Hep C treatment and pregnancy do you wish you knew more about?
- Before we move on to the next section about your practice, do you have any other thoughts to share about the topics we’ve covered so far?

Now we’d like you to think about how you might talk with patients about Hep C treatment in pregnancy.

- What do you think your patients with Hep C know about Hep C treatment?
- If a patient approached you about treatment during pregnancy, how would you respond or manage her care?
- Do you think patients with Hep C are interested in treatment during pregnancy?
  - Why or why not?
  - What issues do you think patients consider when making these decisions/requests?

Next let’s talk about practical issues regarding Hep C treatment. A major issue in Hep C treatment is the importance of linkage to specialty care.

- How do you feel about using prenatal care as a treatment opportunity for Hep C?
- What do you think motivates your patients to remain adherent to any therapy during pregnancy?
- As you may know, Hep C treatment usually involves 8-12 weeks of therapy and could potentially be completed during the second and/or third trimester. Does this alter your perspective?
- Tell us about barriers you perceive to initiating Hep C treatment for pregnant patients.
  - *[Potential probes]: i.e. financial barriers - either patient insurance or provider reimbursement; referral sites – where to send patients, other access issues; lack of safety or efficacy data in pregnancy; lack of professional guidelines or recommendations for treatment in pregnancy; time to counsel and monitor; not sure I understand how treatment works*

**If provider is MFM:** Lastly, we want to ask about research.

- What do you know about current research regarding Hep C treatment in pregnancy?
- What do you see as the gaps in research regarding Hep C treatment in pregnancy?
- How willing would you be to refer your pregnant patients for Hep C treatment trials?
- What barriers do you see to conducting research on Hep C treatment in pregnancy?

Thank you for your participation. Do you have any final comments or questions?

Would you like any references about Hep C? We have some with us if you would like to take them with you. *Interviewer provides 3 take-away points and publications if requested by interviewee.*

**PATIENTS**

**Introduction** *(consent to have been done in advance)*

Thank you for joining us today. You have been asked to participate in this interview because you are a woman with hepatitis C.

In order to expand our knowledge of how to best care for women with hepatitis C, we would like your feedback regarding your knowledge and experiences. I will ask you some questions on your experiences, your familiarity with hepatitis C and its treatment, and your opinions on these issues as related to pregnancy.

Before we begin, I just want to remind you that there are no wrong answers. We are only trying to get a better understanding on how to best care for pregnant women with hepatitis C. We welcome all perspectives and we very much appreciate your time and willingness to share your expertise.

The information you share today will remain completely confidential. Everything you say will be audio recorded, transcribed, and stored on a password-protected computer. Please do not use your name or other identifying information in this interview - if your name or other identifying information is mentioned in the interview, we will not use it in reports. You can choose to participate and to stop participating at any time. Do you have any questions?

**Part 1: Your background**

Let’s start with a few questions about yourself.

- About how long ago were you diagnosed with hepatitis C?
  - What type of appointment were you in when you learned about your diagnosis? (i.e. prenatal care, routine primary care, other)
    - *[Potential probe]: What type of doctor diagnosed you with Hepatitis C?*
  - What did you learn about your treatment options at that time?
    - What did you think about those treatment options?
- Have you ever been pregnant, including currently?

**If yes:**

- - Tell us about what happened with each pregnancy you had in the last 5 years.
    - *[potential probe]: i.e. live birth, fetal anomaly, neonatal demise, child with congenital infection*
  - Tell us how each child did and is doing now
  - Did you know if you had Hepatitis C in any of those pregnancies?
  - (if live births) Do you know if any of your children have Hepatitis C?
  - Are you pregnant now?

**If yes:**

- - - Do you think this pregnancy has been easier, harder, or similar in difficulty to other pregnancies you have had, or to pregnancies that other women you know have had?
- **If eve**r **pregnant:** Women vary a lot in how they change their behavior while pregnant according to what their doctor says. On a scale of 1-10, where 10 is changing your behavior according to your doctor’s advice and 1 is not changing any behavior at all, how you would you rate how you’ve changed your behavior since you’ve become pregnant, or during your previous pregnancies?
  - Can you tell me about how you decide what behaviors you want to change or not change?
- Do you have any ongoing or recent medical problems that you took medication for?
  - *[probe if no response]: i.e. migraines, HIV, Hepatitis B, allergies?*
- **If ever pregnant:** Can you think of a time when you thought about taking a treatment for a medical condition while you were pregnant?
  - *[Potential probes]: i.e. for migraines, HIV, Hepatitis B, allergies?*
  - **If yes:** What kind of things did you think about when you were deciding to receive the treatment or not?
- **If currently pregnant:** Are you taking any medications currently?
- **If previously pregnant:** Did you take any medications during your prior pregnancies?

**Part 2: Knowledge and Attitudes**

Next, we’d like to ask you about your thoughts regarding hepatitis in pregnancy. Please remember, there are no right or wrong answers during this interview, we are really interested in hearing your honest thoughts and opinions.

- Tell me what you’ve heard about Hepatitis C treatment.

*[Potential probes]:*

- - *Have you ever talked with a medical provider, such as a doctor or nurse, about Hepatitis C treatment?*
  - *What do some of your friends or family members think about treatment?*
  - *How did you get information about Hepatitis C treatment before pregnancy?*
  - *What would you say to other people with Hepatitis C about treatment?*
  - *How do you take Hepatitis C treatment?*
  - *How often would one take the treatment?*
  - *How long does treatment take?*
  - *How well does it work or not work?*
  - *Are there any risks of treatment? What are the risks?*
  - *Are there any benefits of treatment? What are the benefits?*
  - *Do you know the names of any of the treatments?*
  - *How much does treatment cost?*
- **If ever pregnant:** Did anyone ever talk with you about Hepatitis C treatment while you were pregnant? Who?
- **If ever pregnant:** Did anyone ever talk with you about Hepatitis C treatment before you were pregnant? Who?
- Have you ever received treatment for Hepatitis C?

**If yes:**

- - What type of treatment was it?
  - Tell us about your experience with the treatment. How did it go?
  - Did you complete the treatment, or did you not complete the treatment?
  - Did you have any complications from the treatment? If yes: What types of complications did you have?

**If no:**

- - Did you ever try to get treatment for Hepatitis C? What happened when you tried to get treatment?
    - *[Potential probe]: Did you ever try to get treatment and you were not able to? What happened?*
- How big of a deal do you think having Hepatitis C is for someone during their pregnancy?
- How big of a deal do you think having HIV is for someone during their pregnancy?
- Have you ever known anyone else with Hepatitis C?
  - **If yes**: Have any of them received treatment?

**If yes:**

- - - - Has this person ever talked to you about their Hepatitis C treatment? What kinds of things have they told you?
      - Has this affected you or your opinions about Hepatitis C treatment? How?
- Let’s say you decided you wanted treatment for Hepatitis C. How would you go about trying to get it?
- Tell us what you think about taking medication for Hepatitis C treatment during pregnancy.

*[Potential probes]:*

- - *What are reasons you may want to take medication for Hepatitis C treatment during pregnancy?*
  - *What are reasons you may not want to take Hepatitis C treatment during pregnancy?*
  - *If you were pregnant and your doctor recommended Hepatitis C treatment during your pregnancy, would you be open to taking the medication, or would you not be open to taking the medication? Why or why not?*
  - *What questions might you have for your doctor if they recommended Hepatitis C treatment during pregnancy?*
- If you were making a decision about whether or not to take medication for Hepatitis C treatment during your pregnancy, who would you want to talk with about this decision?
  - - *[Potential probe]: obstetric provider, different specialist, friends, family, partner, others*
  - Who would not want to talk to about this decision?
  - Would you look anywhere else for information? Where would you look?
    - *[Potential probe]: for example, the internet? What web sites would you look for?*

Lastly, we’d like to know your thoughts on treatment-related research. Remember, there are no right or wrong answers during this interview, we are just looking for your honest opinion.

- Do you have any thoughts about research on Hepatitis C during pregnancy?

*[background info in case participant asks] Current research suggests treatment may be safe, but more research needs to be done on this issue during pregnancy. If research does not occur, treatment may not become widely available for pregnant women.*

- What do you think about pregnant women participating in research in general?
- Have you been in any other research studies, either during or before pregnancy?
- If you were approached about participating in a research study involving Hepatitis C treatment, what are the factors that are important to you when deciding whether or not to participate?

*[Potential probes]*

- - *What would be reasons to participate in the research? (i.e. your own personal benefit, benefit to your fetus, benefit to society and other future patients, incentives to participate such as free treatment or money)*
  - *What would be reasons to not participate in the research?* *(i.e. logistics, time, or scheduling; unknown benefit to yourself or fetus; unknown risks to yourself or fetus; your thoughts about being “experimented” upon; the potential side effects; suspicious of drug companies or other parties; don’t think it’s necessary; stigma about either having Hepatitis C or having treatment for Hepatitis C; partner or family might not like you being in research; having to answer personal questions)*

Before we wrap up, we have one more question we would like to ask you.

- Is there anything that you would tell other pregnant women who have Hepatitis C?

Thank you for your time and for sharing your thoughts on this important topic with us. Is there anything else you would like to tell us, or do you have any questions for me?
